# Misdiagnosis of cystic tuberculosis of the olecranon

**DOI:** 10.1007/s00132-017-3401-y

**Published:** 2017-02-24

**Authors:** Conglin Ye, Xiaoquan Hu, Xiaolong Yu, Jin Zeng, Min Dai

**Affiliations:** Department of Orthopedics, The First Affiliated Hospital of Nanchang University. Artificial Joints Engineering and Technology Research Center of Jiangxi Province, Nanchang, 330006 Jiangxi China

**Keywords:** Diagnostic errors, Tuberculosis, Olecranon process, General surgery, Pathology, Fehldiagnose, Tuberkulose, Olekranonerkrankung, Allgemeinchirurgie, Pathologie

## Abstract

Osteoarticular tuberculosis accounts for only 1–2% of all cases of tuberculosis, and tuberculosis of the olecranon is extremely rare. In the present study, we describe a case of a 54-year-old woman with cystic tuberculosis of the olecranon, which was initially misdiagnosed as a malignant tumor. The patient subsequently underwent regular antituberculosis treatment and autogenous bone graft, which resulted in relief of all symptoms.

## Zusammenfassung

Die osteoartikuläre Tuberkulose macht nur 1–2 % aller Tuberkulosefälle aus, eine Tuberkulose des Olekranons ist extrem selten. In dem vorliegenden Beitrag wird der Fall einer 54-jährigen Frau mit zystischer Tuberkulose des Olekranons beschrieben, bei der anfänglich die Fehldiagnose eines malignen Tumors gestellt worden war. Bei der Patientin erfolgte daraufhin eine regelmäßige tuberkulostatische Behandlung mit autologer Knochentransplantation, darunter kam es zur vollständigen Besserung der Symptome.

## Introduction

Mycobacterium tuberculosis may involve any part of human body [1, 2]. The spinal column, femur, tibia, and fibula are the most common areas involved in skeletal tuberculosis [3, 4]. Osteoarticular tuberculosis constitutes only 1–2% of all cases of tuberculosis [5] and has remained a diagnostic mystery particularly when the disease affects unusual sites [6]. The current study presents a case of a 54-year-old woman with cystic tuberculosis of the olecranon, which is an extremely rare site for bony tuberculosis.

## Case report

A 54-year-old woman presented with painful swelling of the right elbow. She stated that the swelling appeared about 2 months ago and had been increasingly aggravated during the disease process. A week previously, the patient had been diagnosed with a malignant tumor by a local hospital. Thus, she came to our department to confirm the diagnosis. According to her, about 1 year ago, a 5 × 3 cm mass in the chest wall was detected and she was diagnosed with chest wall tuberculosis. Later she underwent surgery to remove the nodule and was told to take the postoperative antitubercular agent consecutively for 2 months. However, she quit taking the medicine midway. She had no related history of injury, concomitant diseases, or family history except the tuberculosis of chest wall. In the general physical examination, no limited motion in the right elbow was observed. The skin of the right elbow was normal without any erythema or ulcer. However, there were pain, swelling, and slight paraesthesia in the right upper extremity. There was no fever or respiratory sounds accompanying the pain. The pain was more severe at night. Remarkably, the patient lost 5 kg during the disease process.

**Fig. 1:**
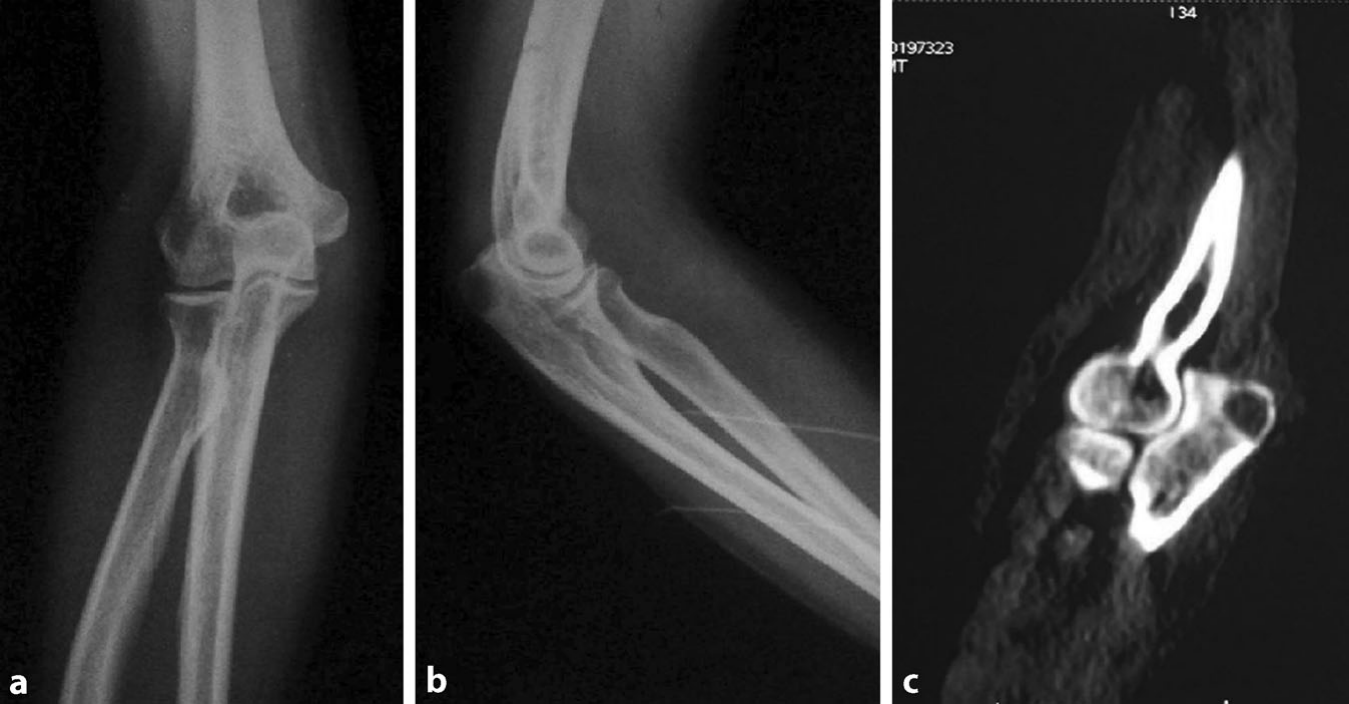
**a** Anteroposterior plain radiograph of the right elbow indicated that there was a osteolytic lesion in the upper back end of olecranon. **b** Lateral plain radiograph showed that there was a poorly defined osteolytic lesion in the upper back end of olecranon. **c** Computed tomography scan revealed a poorly defined osteolytic lesion in the upper back end of olecranon

**Fig. 2:**
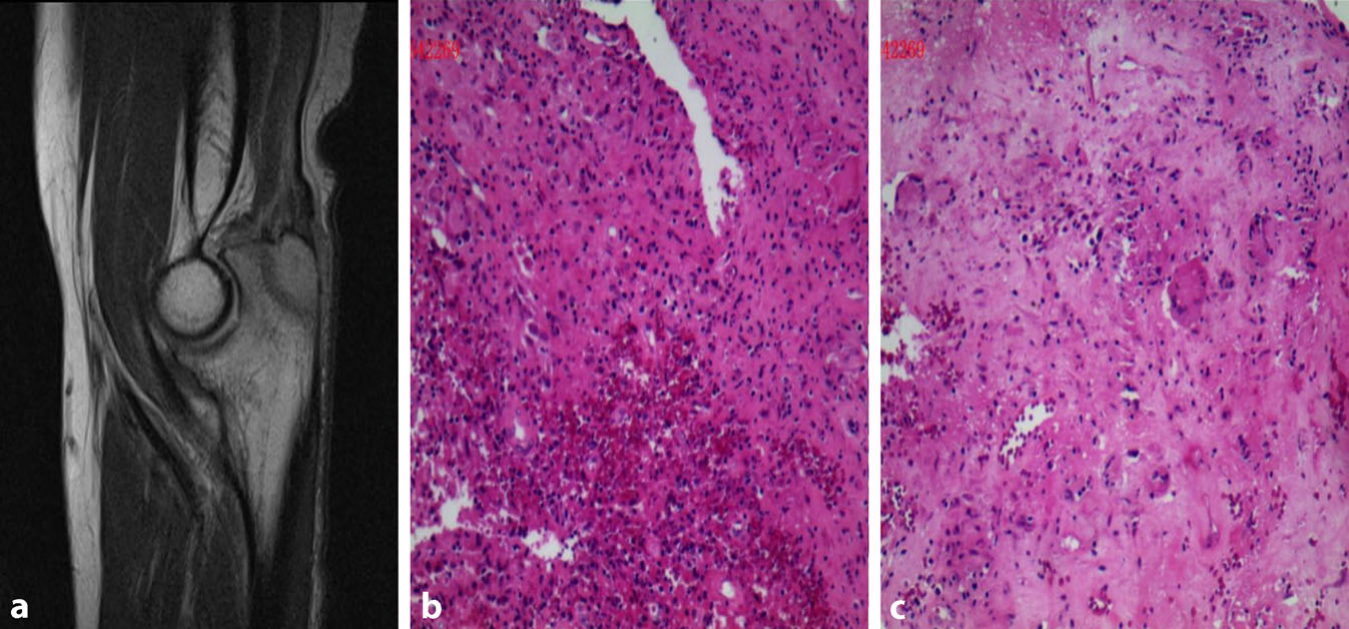
**a** MRI of right elbow revealed a slightly long T1, long T2 signal of a size of 7 × 0.9 cm in the rear end of right olecranon and its partial edge was hazy. **b, c** The histological findings showed typical epithelioid granuloma in a background of marked inflammation comprising of sheets of neutrophils, histiocytes, plasma cells, and increased lymphocytic infiltration. Specific stains showed acid-fast staining (+) and periodic acid Schiff stain (PAS) (−) (magnification, ×200)

In a further examination, no soft tissue mass or bony prominence was found over the right elbow, and the test for elbow instability was negative. Physical examination showed no palpable head, neck, supraclavicular, axillary, or epitrochlear lymph nodes. In addition, many different blood examinations were performed, including full blood check (FBC), erythrocyte sedimentation rate (ESR), C reactive protein (CRP), rheumatoid factor (RF), and anti-cyclic cirullinated peptide antibodies (anti-CCP) and blood culture. According to laboratory findings, FBC was normal, and CRP and ESR were 70 mg/l and 35 mm/h, respectively (normal range: CRP: 0–8 mg/l; ESR: 0–20 mm/h). RF, anti-CCP, and blood culture were all negative. The HIV test was negative. The computed tomography (CT) scan of the chest revealed no pulmonary nodules or other abnormal signs. Plain radiographs indicated that there was a poorly defined osteolytic lesion in the upper back end of olecranon (Fig. 1a, b). A CT scan of the right elbow revealed irregularly osteolytic lesions in both the intercondylar bone and the upper back end of olecranon (Fig. 1c). MRI of right elbow revealed a slightly long T1, long T2 signal of a size of 7 × 0.9 cm in the rear end of right olecranon and its partial edge was fuzzy (Fig. 2a).

The histological findings after open surgical biopsy showed typical epithelioid granuloma in a background of marked inflammation comprising of sheets of neutrophils, histiocytic, plasma cells, and increased lymphocytic infiltration. Specific stains showed acid-fast staining (+) and periodic acid Schiff stain (PAS) (–) (Fig. 2b, c). All the findings above were consistent with tuberculosis. Therefore, the diagnosis of tuberculosis of the olecranon was made.

Antituberculosis chemotherapy was began immediately the moment she was diagnosed with cystic tuberculosis of the olecranon. After taking antituberculosis agents for 4 weeks, her blood routine was reviewed. Fortunately, ESR and CRP were normal and symptoms had disappeared.

The patient was discharged without any further complications. At the 3‑month follow-up, the patient was absolutely symptom free and had returned to work.

## Discussion

Skeletal tuberculosis is an unusual disease when compared with pulmonary tuberculosis, and it is estimated that skeletal tuberculosis involves about 10–15% of all tuberculosis patients [7, 8]. Although the spine is considered to be the most common site of skeletal tuberculosis, no bone is immune to the bacilli. Nevertheless, the olecranon is indeed an extremely rare site for bony tuberculosis. In most cases, skeletal tuberculosis is determined late since tuberculosis lesions are paucibacillary and smears are usually negative [9, 10].

Notably, diagnosis of skeletal tuberculosis is difficult due to its indolent nature lacking of any specific signs, symptoms, or radiographic findings. Although raised ESR and positive Monteux test are consistent findings, these are not diagnostic basis of tuberculosis in endemic areas. Radiographic findings in tubercular osteomyelitis include radiolucent lesion with irregular margin and surrounding sclerosis. The cystic cavitary lesions on radiographs are highly nonspecific and simulate with pyogenic osteomyelitis, fungal infection, metastasis, telangiectactic osteosarcoma, aneurysmal cyst, sarcoidosis, eosinophilic granuloma, or chordoma [5, 11, 12]. Bone lesions may present in a bizarre fashion and mimic other diagnoses. Lack of familiarity with and awareness of cystic tuberculosis may lead to delay or errors in diagnosis.

In the present study, the patient was mistakenly diagnosed with a malignant tumor in a local hospital, which resulted in delayed treatment. There were no tuberculous toxic syndromes such as fever, night sweat, or hypodynamia before or after the onset of illness. Instead, the patient had the history of obvious night pain and rapid weight loss. In addition, SPECT whole body bone imaging showed that there were increased uptake foci of nuclide distributing abnormally in the left zygomatic arch, the 7th rib in left rear, bilateral elbow joints, right knee joint, and right instep bones. Hence, it was very likely to be misdiagnosed as a bone tumor rather than tuberculosis before the open surgical biopsy.

The disease was determined based on the patient’s physical condition, clinical examination, serological evaluation, X‑ray, microbiological, and histological findings. A definite diagnosis of osteoarticular tuberculosis is given through the isolation of mycobacterium tuberculosis from the skeletal site. It is often done through bone and synovial biopsy. Therefore, open surgical biopsy of bone tissue is the golden standard to detect osseous tuberculosis. It was reported that that confirmation of musculoskeletal tuberculosis is solely based on identification of epithelioid granuloma and caseous necrosis or tubercle bacilli in open surgical biopsy or tissue culture studies. In the present case, we performed open surgical biopsy rather than fine needle biopsy because the former is more accurate and intuitive. However, the local hospital did not conduct a biopsy, which led to the misdiagnosis.

Following surgical treatment and regular antituberculosis treatment, our patient was absolutely symptom free at the 3‑month follow-up. In consideration of the chance of recurrence, the authors suggest long-term follow-up for these kinds of patients.

## Conclusion

Tuberculosis located in an unusual location is quite likely to be misdiagnosed as a tumor due to the similar clinical manifestations and imaging findings, which might cause delayed treatment, excessive medical treatment, or even worse consequences. However, biopsy especially open surgical biopsy is very helpful in distinguishing between these two diseases. Disease history of tuberculosis is also of great importance. The current research reminds us that we should be much more cautious about differential diagnoses.
